# Antiplatelet therapy with CABG: chaos in the Netherlands

**DOI:** 10.1007/s12471-017-1022-z

**Published:** 2017-07-13

**Authors:** F. W. A. Verheugt

**Affiliations:** Department of Cardiology, Onze Lieve Vrouwe Gasthuis, Amsterdam, The Netherlands

One of the factors affecting the fate of arterial and venous bypass grafts in coronary artery bypass grafting (CABG) is optimal antithrombotic protection. Traditionally, antiplatelet therapy is the treatment of choice to protect grafts against occlusion [1]. Oral anticoagulants have shown to be effective as well [2], but not superior to antiplatelet agents [3]. Therefore, aspirin has become the standard of care in the prevention of graft occlusion [4]. Nowadays, a complicating factor is the use of dual antiplatelet therapy (DAPT) with aspirin and a P2Y12 blocker (clopidogrel, prasugrel or ticagrelor) in patients who have recovered from acute coronary syndromes (ACS) [5, 6]. Not only do they have to undergo CABG for their index ACS, they may already be on DAPT when they become eligible for CABG. These considerations make the choice for antithrombotic protection in patients undergoing CABG complex.

In this issue of the *Netherlands Heart Journal*, Janssen et al. present the results of a survey amongst the cardiothoracic centres in the Netherlands on their strategies of perioperative antithrombotic therapy in CABG in the Netherlands [7]. The bottom line is that there is a large variation in the use of antithrombotic therapy around CABG, which was quite similar to the situation 27 years ago [8]. Furthermore, they show the results of a registry in their own centre which actually confirmed the disturbing findings in the rest of the country. The authors correctly conclude that this is due to the lack of stringent guidelines on antiplatelet therapy in the field of coronary surgery.

With regards to preoperative aspirin, the majority of centres discontinue aspirin, which is not unanimously advised by the international guidelines (see below). In ACS patients P2Y12 blockers are discontinued between 4 and 7 days preoperatively in most centres, as mandated by the guidelines. Astonishingly, the majority of centres (including the reporting one) do not restart P2Y12 blockers after surgery in ACS patients. This is against the guidelines, because the benefit of restarting was already shown in CURE, the mother of all ACS trials on P2Y12 blockers [9].

There are few guidelines on the perioperative management of platelet inhibition in CABG. The most specific guideline from Europe [4] dates from 2008, and the American one is from 2014 [10]. The 2014 guideline issued by the European Association for Cardio-Thoracic Surgery [11] is not clear on this issue and focusses more on percutaneous revascularisation than CABG. A simplified recommendation for antiplatelet management in CABG is distilled from these guidelines and is given in Tab. 1.

**Table 1:** Recommendation with level of evidence of timing and duration of antiplatelet therapy peri-CABG

			CABG indication	
Timing	Agent	Stable CAD without stent(s)	Stable CAD with recent stent(s)	ACS with or without stent(s)
Preoperative	Aspirin P2Y12	Continue (IA)^a^ n.a.	Continue (IA) Stop 5–7 days (IIA)	Continue (IA) Stop 5–7 days (IIA)^b^
Postoperative	Aspirin P2Y12	Lifelong (IA) n.a.	Lifelong (IA) Day 1 restart (IIA)^c^	Lifelong (IA) Day 1 restart (IIB)^d^

*n.a.* not applicable

^a^Unless expected high perioperative bleeding risk

^b^Unless continued clinical instability

^c^Duration depending on stent type and patient characteristics

^d^Duration at least 1 year after index ACS

In conclusion, even in 2017 there is little consensus on antiplatelet strategies around CABG in the Netherlands. This is probably due to the rather vague international guidelines, which is a consequence of a relative paucity of evidence from randomised clinical trials. Because of the good results of cardiac surgery in the Netherlands, cardiothoracic centres should continue to follow their own clinical practice in the antiplatelet management in CABG.
