# Erratum to: Antiplatelet therapy with CABG: chaos in the Netherlands

**DOI:** 10.1007/s12471-017-1031-y

**Published:** 2017-08-09

**Authors:** F. W. A. Verheugt

**Affiliations:** Department of Cardiology, Onze Lieve Vrouwe Gasthuis, Amsterdam, The Netherlands

**Erratum to:**

**Neth Heart J 2017**

**DOI** 10.1007/s12471-017-1022-z

In the PDF version of the article, Tab. 1 was incorrectly displayed. The corrected version is shown below.

**Table 1:** Recommendation with level of evidence of timing and duration of antiplatelet therapy peri-CABG

			CABG Indication	
Timing	Agent	Stable CAD without stent(s)	Stable CAD with recent stent(s)	ACS with or without stent(s)
Preoperative	Aspirin	Continue (IA)^a^	Continue (IA)	Continue (IA)
	P2Y12	n.a.	Stop 5–7days (IIA)	Stop 5–7 days (IIA)^b^
Postoperative	Aspirin	Lifelong (IA)	Lifelong (IA)	Lifyelong (IA)
	P2Y12	n.a.	Day 1 restart (IIA)^c^	Day 1 restart (IIB)^d^

*n.a.* not applicable

^a^Unless expected periperative high bleeding risk

^b^Unless continued clinical instability

^c^Duration depending on stent type and patient charcteristics

^d^Duration at least 1 year after index ACS

